# New-Onset Atrial Fibrillation in Adult Patients After Cardiac Surgery

**DOI:** 10.1007/s40140-019-00321-4

**Published:** 2019-04-24

**Authors:** Peter S. Burrage, Ying H. Low, Niall G. Campbell, Ben O’Brien

**Affiliations:** 1 Department of Anesthesiology, Dartmouth-Hitchcock Medical Center, Lebanon, NH 03756, USA; 2 Department of Cardiology, Wythenshawe Hospital, Manchester, UK; 3 William Harvey Research Institute, Queen Mary University of London, St Bartholomew’s Hospital, London, UK; 4 Outcomes Research Consortium, Cleveland Clinic, Cleveland, OH, USA

**Keywords:** Atrial fibrillation, Cardiac surgery, Adult, Risk factors, Prevention, Treatment

## Abstract

**Purpose of Review:**

An overview of recent literature regarding pathophysiology, risk factors, prophylaxis, and treatment of new-onset atrial fibrillation (AF) in post-cardiac surgical patients.

**Recent Findings:**

AF is the most frequent adverse event after cardiac surgery with significant associated morbidity, mortality, and financial cost. Its causes are multifactorial, and models to stratify patients into risk categories are progressing but a consistent, evidence-based system has not yet been developed. Pharmacologic and surgical interventions to prevent and treat this complication have been an area of ongoing research and recent societal guidelines reflect this.

**Summary:**

Inconsistencies remain surrounding how to best identify higher-risk AF patients, which interventions should be used to prevent and treat AF, and which patient groups should receive these interventions. The evidence for these available strategies and their place in contemporary guidelines are summarized.

## Introduction

Atrial fibrillation after cardiac surgery (AFACS) is the most common postoperative complication following cardiac surgical procedures and occurs in 25% after isolated coronary artery bypass grafting (CABG), 30% after isolated valvular procedures, and 40–50% following combination CABG/valvular operations [1]. Notably, the incidence of AFACS has remained largely unchanged despite contemporaneous improvements in cardiac surgery-associated morbidity and mortality [2, 3].

While postoperative atrial fibrillation (POAF) is not a problem unique to cardiac surgical patients, rates of AFACS are significantly higher than those in both thoracic surgery (10–30%) and non-cardiac, non-thoracic surgery (1–15%) [4, 5].Additionally, AFACS has different characteristics when compared to POAF following non-cardiac surgery including potential mechanisms and data supporting measures for prevention and treatment.

While AFACS may have once been considered a transient and predominantly benign complication, its associations with increased morbidity such as postoperative stroke, sternal and respiratory tract infections, and gastrointestinal dysfunction and renal dysfunction as well as increased short- and long-term mortality are now well-established [6–9]. The onset of AFACS has also been correlated with longer, and costlier, lengths of stay (LOS) in the intensive care unit and hospital, in addition to increased rates of readmission [7, 10–12]. These outcomes translate into a substantial financial impact; approximately $2 billion annually has been attributed to AFACS care specifically [13], out of a total annual expenditure related to AF care in the USA of more than $6 billion, [13–16]. It is uncertain to what extent these relationships are causal.

Taking into account the considerable effect that improvements in AFACS care could have on both patient outcomes and financial healthcare burdens, substantial research efforts have been directed at identifying the mechanisms behind AFACS as well as effective prophylactic and treatment strategies for this adverse postoperative event. The goal of the review article is to highlight the current understanding regarding AFACS pathogenesis, risk factors, prophylaxis, and treatment. To this end, on October 17, 2018, the following search operations were performed in PubMed: (((“Cardiac Surgical Procedures”[Mesh] OR cardiac surg*[ti] OR after cardiac surgery[tiab] OR coronary artery bypass[ti] OR cabg[ti] OR coronary artery surgery[ti] OR heart valve*[ti] OR mitral valve*[ti] OR aortic valve*[ti]) AND (“Atrial Fibrillation”[Mesh] OR afib[ti] OR “a fib”[ti] OR Atrial Fibrillation*[ti] OR “AF”[ti]) AND (etiology OR postoperative OR new onset[tiab])) NOT (“Comment” [Publication Type] OR “Letter” [Publication Type] OR “Editorial” [Publication Type]) AND (“2015/10/17”[PDat] : “2018/10/17”[PDat] AND English[lang])) and relevant publications were identified.

## AF Pathophysiology and Mechanisms

AF is a supraventricular arrhythmia characterized by erratic atrial depolarizations leading to disorganized, ineffective atrial contractions, and variable atrioventricular nodal conduction, which results in an irregular ventricular rate [17]. An expert consensus document defined the diagnosis of AF as requiring a 12-lead electrocardiogram (ECG) or a rhythm strip of at least 30-s duration that demonstrates (1) irregular RR intervals in the absence of complete AV block, (2) an absence of distinct P waves on surface ECG, and (3) an atrial cycle length that is variable and generally less than 200 ms [18].

It is unlikely that there is a single unifying mechanism behind the development of AF, but it is generally agreed that AF requires both a trigger and a susceptible atrial substrate that allows for maintenance of the arrhythmia [19, 20]. Most commonly, this trigger impulse is thought to arise from the myocardial sleeves of the left atrium that blend into the approaching pulmonary veins [21]. Histologically, this area is remarkable for a relatively unique myocardial fiber structure that has areas of discontinuity and fibrosis. This particular tissue architecture may be responsible for the electrophysiologic properties conducive to the generation of frequent ectopic foci that can act as a trigger for AF initiation [21]. Notably, there are multiple ganglionated autonomic nerve plexuses that are associated with these left atrium/pulmonary vein junctions which provide an anatomical basis for the development of spontaneous ectopic foci by variations in sympathetic and parasympathetic tone [22]. Interestingly, an episode of atrial fibrillation lasting only hours to weeks can lead to electrophysiological remodeling mediated by alterations in function of several ion channels, most commonly those responsible for calcium and potassium fluxes. A duration of months or longer can then lead to progressive structural remodeling of the atrium itself, heralded by progressive fibrosis, dilation, and hypertrophy. Together, these changes can act in a feed-forward manner to further promote a pro-arrhythmic substrate [23, 24].

Patient factors, cardiac surgical factors, and endogenous/exogenous postoperative factors may align to specifically predispose patients presenting to the cardiac surgical operating room for developing AFACS. Patients requiring cardiac surgery frequently have pre-existing risk factors for atrial dilation including hypertension, myocardial ischemia, and valvular abnormalities such as mitral regurgitation. Perioperatively, direct surgical trauma associated with atriotomy incisions and pericardial disruption may also contribute to local inflammation and subsequent alterations in atrial electrical excitability. It has also been observed that while on cardiopulmonary by-pass, the atria can remain electrically active despite sufficient cardioplegia administration for ventricular electrical arrest. This continuing activity may predispose the atria to ischemia and subsequent arrhythmias [25]. Large fluid shifts perioperatively and electrolyte disturbances may also be contributory [13]. In the postoperative period, the patient may be exposed to a number of proarrhythmogenic factors, including increased endogenous catecholamines, inflammatory and oxidative mediators secondary to surgical stress and the systemic response to cardiopulmonary bypass, use of exogenous catecholamines for inotropic support, and variations in both intravascular volume status and systemic blood pressure leading to changes in atrial stretch and myocardial perfusion, respectively.

Two separate phases regarding the risk of development of AFACS, with distinct associated factors, have been described. The first phase encompasses the first 18 h postoperatively with the greatest risk at hour zero, and the second phase occurs with the risk peaking at 24–48 hrs [26]. This observation raises the possibility that separate mechanisms may be responsible for AFACS development within each phase.

## Risk Factors for AFACS

Different series have reported a number of risk factors for the development of AFACS including a prior history of paroxysmal AF, obesity, chronic obstructive pulmonary disease, chronic renal failure, rheumatic heart disease, and male gender, as well as echocardiographic predictors such as abnormal left ventricular systolic and diastolic function, left ventricular hypertrophy, and increased left atrial volume. The most consistent independent risk factor across multiple studies has been increasing patient age [7, 13, 27–45]. A number of scoring systems have been generated to predict the risk of developing AFACS with the goal of being able to preoperatively identify high-risk patients to allow for use of appropriately targeted prophylactic regimens as recommended by a number of societal guidelines [7, 31–45]. Furthermore, to date, consistent reproducibility of factors between studies has been lacking and a post hoc validation analysis utilizing three risk scores derived from some of the largest trials obtained a low predictive value for these scores when applied prospectively to a patient cohort [46]. It remains speculative whether the identification of a higher risk AF population could ultimately translate to improve patient outcomes, although AFACS rates, and associated increases in length of stay and cost, might as well be reducible with targeted aggressive prevention.

There is significant heterogeneity in the literature regarding how POAF is defined, identified, and reported. Some studies use opportunistic identification of AF (typically retrospective studies) whereas others utilize continuous monitoring that is more likely to identify asymptomatic AF and results in a higher reported incidence. This heterogeneity can make a direct comparison between studies problematic. We would advise that all future prospective studies reporting the incidence of POAF should supplement routine in-patient heart rhythm monitoring with a 5-day continuous Holter recording, allowing the independent confirmation of the diagnosis of AF.

**Fig. 1:**
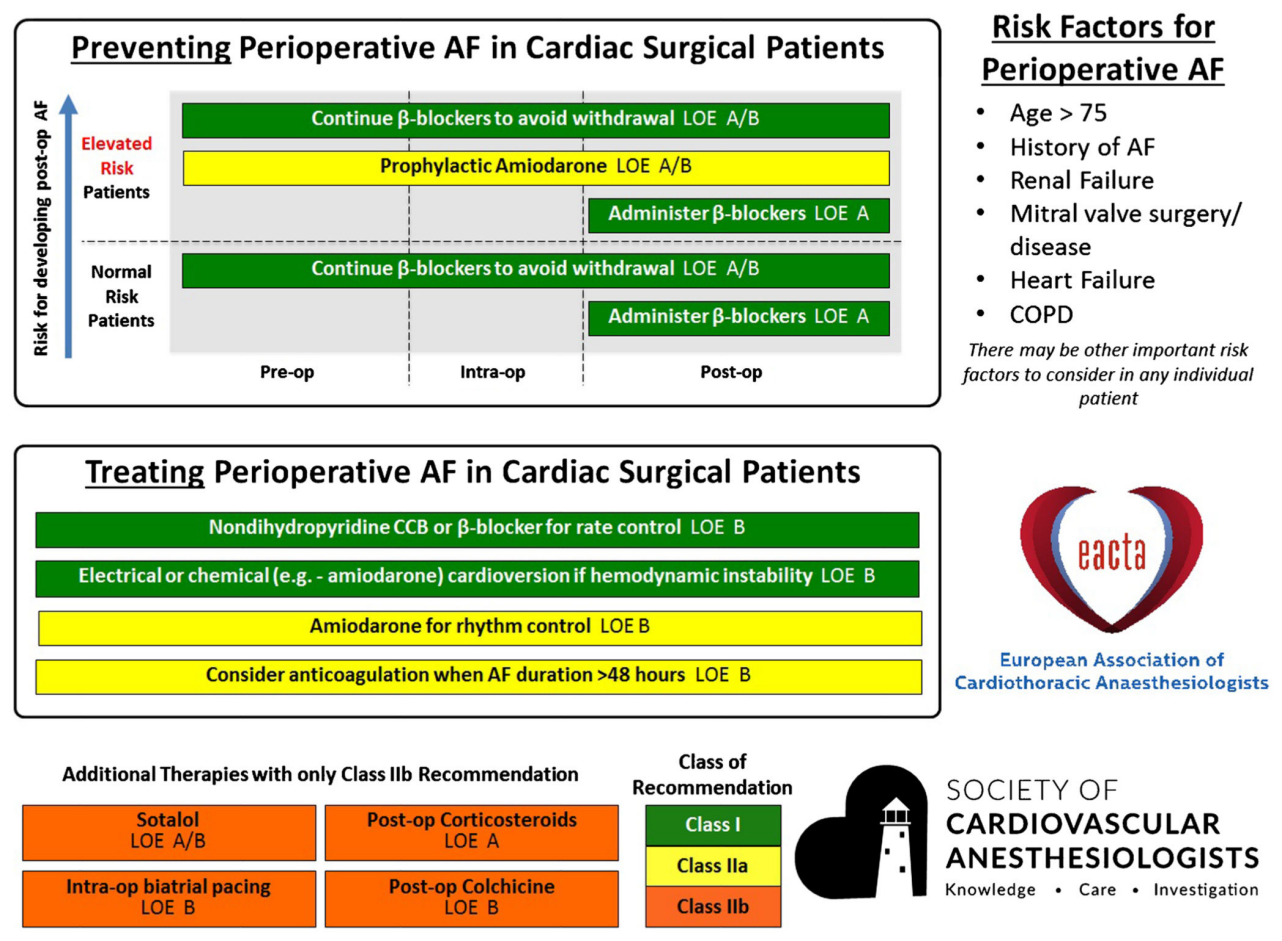
SCA/EACTA Graphical Practice Advisory for the management of AFACS, summarizing evidence-based prevention and treatment strategies and risk factors for perioperative atrial fibrillation in cardiac surgical patients. Reproduced from Muehlschlegel JD, Burrage PS, Ngai JY, Prutkin JM, Huang CC, Xu X et al. Society of Cardiovascular Anesthesiologists/European Association of Cardiothoracic Anaesthetists Practice Advisory for the Management of Perioperative Atrial Fibrillation in Patients Undergoing Cardiac Surgery. Anesth Analg. 2019;128(1):33-42, accessible at https://journals.lww.com/anesthesia-analgesia/Fulltext/2019/01000/Society_of_Cardiovascular.11.aspx, with permission from Wolters Kluwer Health, Inc.

While a number of societies have released guidelines regarding prophylactic strategies for AFACS in high-risk patients, there is not currently a consistent, evidence-based system for the stratification of patients into different risk groups. In this context, the Society of Cardiovascular Anesthesiologists (SCA) and the European Association of Cardiothoracic Anaesthesiologists (EACTA) Clinical Practice Improvement Group for AF after Cardiac Surgery recently published a comprehensive practice advisory, in which they also created a list of AFACS risk factors and prophylactic strategies using expert opinion, based on published risk score models for AFACS. These risk factors and prophylactic and therapeutic strategies have been summarized in a graphical advisory tool (Figure 1) [47••, 48••] and may enable improved adherence to evidence-based recommendations.

## Preventative Strategies and Associated Evidence Base

Many different pharmacologic agents and surgical strategies have been studied for preventing the development of AFACS. Strategies using medical-based interventions have focused on several general areas including optimization of electrolytes, prophylactic use of antiarrhythmic medications, reduction of both systemic and localized inflammation, moderating autonomic influences, reduction of oxidative stress secondary to surgery, and choice of vasoactive medication. Surgical-based therapies that have been investigated have included the use of exogenous pacing, modifications to juxtaposed anatomic structures including the pericardium and the anterior fat pad, addition of a concurrent ablation procedure, and the effect of an on-pump vs off-pump surgical approach. These prophylactic strategies and the associated strength of society recommendation, when available, are summarized in Table 1.

### Pharmacological Strategies

#### Electrolyte Management

##### Magnesium

Low serum magnesium levels are a predictor for AFACS [112, 113], and hypomagnesemia is common in post-cardiac surgical patients [114, 115]. The effect of magnesium may be attenuated in patients on concomitant beta-blockers [52, 114]. Another consequence of hypomagnesemia is a diminished response to potassium supplementation [116]. A 2013 meta-analysis included 21 studies (*n* = 2988) investigating various dose regimens of intraoperative intravenous magnesium administration on AFACS and supraventricular tachycardia, and it found a significant reduction in postoperative atrial fibrillation in the magnesium group compared to controls (16.5% vs 26.2%, OR 0.55, 95% CI 0.41–0.73, *I*^2^ = 51%) [49••]. Careful magnesium repletion is a generally safe practice in patients with hypomagnesaemia and should be considered in all patients without severe renal dysfunction.

##### Potassium

Potassium is also frequently depleted amongst cardiac surgical patients who do not receive supplementation, and hypokalemia has been identified as a risk factor for AFACS, particularly if serum potassium is below the normal range [49, 50, 116, 117]. Practice surveys reveal that it appears to be routine practice in many centers to target serum potassium levels at the upper end of the normal range (4.5–5.5 mEq/L) [50, 51]. However, there is no definitive evidence of its AFACS prophylactic efficacy or impact on clinical outcomes [49••, 50]. A 2016 prospective double-blinded interventional study of 910 cardiac surgical patients who were randomized to a potassium target of 4.0 mmol/L or 4.5 mmol/L using a computer algorithm found no difference in AFACS [118]. The Tight K trial is an ongoing randomized controlled trial (RCT) to examine AFACS in patients randomized to relaxed (≥ 3.6 mEq/LL) vs tight (≥ 4.5 mEq/L) control of serum potassium [50]. Potassium should be supplemented in patients with hypokalemia, but the additional utility of maintaining a high-normal potassium to prevent AF is currently unproven.

#### Antiarrhythmic Drugs

##### Beta-adrenergic blockers

Beta-blockers have been extensively studied for the prevention of AF after cardiac surgery, and their likely mechanism is a decrease in sympathetic tone, which increases atrial refractoriness and decreases the initiation of arrhythmias [113, 114, 119]. A 2013 meta-analysis of 33 RCTs (*n* = 4698) found that preoperative treatment with beta-blockers resulted in a significant reduction in AFACS (16.3% vs 31.7%, OR 0.33, 95% CI 0.26–0.43, *I*^2^ = 55%) [49]. In addition, many patients in the control groups stopped non-study beta-blockers to participate in the study and some authors have suggested that this withdrawal may be an independent risk factor for AFACS [7, 114]. A meta-analysis in 2006 compared studies that withdrew non-study beta-blockers, against those that continued non-study beta-blockers; while both groups found significant reductions in AFACS with their treatment groups, the group that withdrew beta-blockers demonstrated larger effects between groups [52, 114]. Other studies have examined the effects of different types of beta-blockers on AFACS. It has been reported that oral metoprolol is more effective at reducing AFACS than intravenous esmolol, and carvedilol may reduce AFACS more effectively than metoprolol, an effect that may be explained by carvedilol’s oxidative stress–reducing properties [113]. In addition to continuing preoperative beta-blockers, the 2011 ACCF/AHA guideline for coronary artery bypass grafting (CABG) offers specific Class I recommendations to administer beta-blockers for at least 24 hours before CABG to all patients without contraindications to beta blockade, to reduce the incidence or clinical sequelae of AFACS [120]. Beta-blockers receive a Class I recommendation from multiple societies for AF prophylaxis (refer to Table 1).

Once AF has been initiated, multiple beta-blockers have been studied and found to be effective for rate control, although the most commonly used are esmolol and metoprolol [113]. All beta-blockers have some negative inotropic effects, with the ultrashort-acting beta-blocker landiolol potentially having the most limited impact on inotropy [113, 121]. Beta-blockers have a Class I recommendation in multiple guidelines for use in rate control of AFACS (see Table 2).

##### Amiodarone

Amiodarone has predominantly potassium channel–blocking antiarrhythmic properties, but also exhibits some degree of action at the beta-adrenergic receptor, sodium and calcium channels [113]. Amiodarone use is associated with adverse events such as bradycardia and hypotension, as well as potential pulmonary, hepatic, and thyroid toxicity, though rarely with short-term use [49, 114]. Its long-term use requires regular monitoring of liver and thyroid function [123]. Amiodarone is also contraindicated in patients with an accessory pathway and can cause bradycardia and QT-interval prolongation [113]. A 2013 meta-analysis of 33 RCTs (*n* = 5402) found a significant reduction in AFACS in patients who received prophylactic amiodarone compared to that in controls (19.4% vs 33.3, OR 0.43, CI 0.34–0.54, *I*^2^ = 63%)[49]. However, dosage regimens and administration routes, including loading doses and infusion rates, varied between studies [49]. There was a reduction in length of stay for those patients receiving prophylactic amiodarone compared with controls, but no decrease in mortality [49••]. Amiodarone receives a Class IIa recommendation by the ACC/AHA/HRS, ACCF/AHA, ESC guidelines and the SCA/EACTA Practice Advisory for AF prophylaxis.

Amiodarone has also been studied as a treatment for AFACS once it occurs. It is an effective rhythm control agent that also has rate-control properties. Authors of a 2011 database study of dronedarone, amiodarone, sotalol, flecainide, and propafenone in AF patients reported that even though amiodarone was most effective at maintaining sinus rhythm, they found a trend toward higher mortality in patients on amiodarone when compared with the other pharmacological agents [124]. Some authors caution that ruling out intracardiac thrombi by transesophageal echocardiogram (TEE) should be considered before using amiodarone to treat AFACS of 24–48-hrs duration, as discussed further below under “Anticoagulation” [113]. As noted in Table 2, amiodarone has a Class IIa recommendation from the SCA/EACTA Practice Advisory for use in the treatment of AFACS.

##### Sotalol

Sotalol is an antiarrhythmic drug with both beta-adrenoreceptor and potassium channel–blocking activity [68]. Its utility is limited due to the risk of significant bradycardia, QT prolongation, and ventricular arrhythmias including torsades de pointes, particularly in patients with electrolyte disturbances [116, 119]. A 2013 meta-analysis of 11 studies (*n* = 1609) concluded that sotalol was associated with a significant reduction in AFACS compared with that of controls (18.1% vs 40.0%, OR 0.34, 95% CI 0.26–0.43, *I*^2^ = 3%) [49••]. However, many of the trials in this meta-analysis used beta-blockers instead of placebos in the control groups [114]. Another meta-analysis which specifically compared patients receiving sotalol to those receiving beta-blockers reported that the sotalol group had a decrease in AFACS compared to the beta-blocker group (OR 0.42, 95% CI 0.26–0.65) [52, 114]. Sotalol receives Class IIb recommendations by multiple societies for AF prophylaxis in high-risk patients (refer to Table 1).

Sotalol has been well-established for the pharmacological conversion and maintenance of sinus rhythm in the general population with AF [125], but has been less well-studied specifically in the setting of AFACS treatment. Since many AFACS treatment options have been adapted from treatment of AF in general, it would be reasonable to extrapolate its use to the cardiac surgical population as well. Its use in the postoperative setting might be limited by any hypotension and renal impairment, particularly in patients with heart failure, and like many other antiarrhythmic agents, should include close monitoring of serum electrolyte levels and the QT interval (3).

##### Ranolazine

Ranolazine received FDA-approval as an anti-anginal medication in 2006 and has an acceptable safety profile even in patients with structural heart disease [126]. It has antiarrhythmic effects resembling that of amiodarone, inhibiting inward sodium and rectifying potassium channels, resulting in prolonged effective refractory period in the atria [126]. Ranolazine has been found to be an effective rhythm control strategy with few adverse events in the general population with AF [126]. In cardiac surgical patients, two recent meta-analysis of the same 4 studies (all single center, 2 retrospective, 1 prospective, 1 randomized trial) have been published, and both report that starting ranolazine preoperatively was associated with a significant reduction in AFACS events, with one meta-analysis reporting 13% AFACS in the ranolazine group compared with 32% in controls [72], and the other a risk ratio of 0.44 ((0.25, 0.78), *p* = 0.005) [73]. The authors note that although the pooled treatment effect of a greater than 50% risk reduction appears impressive, they cannot make definite conclusions due to the small number of studies and heterogeneity in ranolazine dose regimens [73]. Ranolazine’s potential role in AF prophylaxis has not yet been addressed by specific guidelines, but it appears to hold some promise.

##### Non-dihydropyridine Calcium Channel Blockers

Calcium channel blockers are a potential alternative in patients where beta-blockers are contraindicated [114]. However, most of the evidence for their use pertains to heart rate control in patients with AF and there is little evidence to support their prophylactic use to prevent AFACS. They are contraindicated in patients with left ventricular impairment, which may limit their use in this patient cohort. While a 2003 meta-analysis of RCTs found that the preoperative, intraoperative, or early postoperative (within 48 hrs) use of non-dihydropyridine calcium channel blockers was associated with a significant decrease in supraventricular arrhythmias, adverse effects in the form of increased atrioventricular blocks and low cardiac output syndrome limit their use [114]. This class of agents is not referenced for prophylaxis in any society guidelines to date.

In the AFACS treatment setting, verapamil and diltiazem are more often used in patients who have contraindications to beta-blockers, or in conjunction with beta-blockers [113]. RCTs comparing beta-blockers to non-dihydropyridine calcium channel blockers have found that the calcium channel blockers are less effective for rate control when used as the sole agent and are associated with more hypotension [121, 127], which is more pronounced with verapamil [113]. Of note, these are also contraindication in patients with an accessory pathway [113]. Calcium channel blockers receive a Class I recommendation from multiple societies for their use in AFACS rate control (See Table 2).

#### Anti-inflammatory Agents

##### Corticosteroids

A 2011 meta-analysis of 14 RCTs (*n* = 1974) found that steroid prophylaxis was effective against AFACS (25.1% vs 37.3% in controls, OR 0.56, 95% CI 0.44–0.72, *p* < 0.0001). There was significant heterogeneity amongst the studies regarding the type of steroid received: methylprednisolone (51.4%), dexamethasone (34.3%), hydrocortisone (5.7%), prednisolone (2.9%), or a combination of methylprednisolone and dexamethasone (5.7%) [74]. The authors also observed that steroid administration was not associated with an increased risk of postoperative infection, need for re-exploration, or mortality [74].

A 2009 meta-analysis of RCTs (*n* = 1046) aimed at determining the impact of different corticosteroid regimens concluded that overall corticosteroid use is associated with a reduced risk of AFACS and this effect became more prominent when low-dose and very high-dose steroid studies were excluded (OR 0.32, 95% CI 0.21–0.50, *p* < 0.00001) [75]. They concluded that a single dose of moderate-dose hydrocortisone should be considered at induction for the prevention of AFACS in high-risk patients. The authors of a 2018 meta-analysis of 56 RCTs (*n* = 16,013) echoed the overall findings that new-onset AFACS was lower in the steroid group (25.7% vs 28.3%, RR 0.91, 95% CI 0.86–0.96, *p* = 0.005, *I*^2^ = 43%), but cautioned that this effect was driven by only small trials, and larger trials showed no effect [76].

The optimal dose of steroids, interval and total therapy duration has yet to be established. Perioperative use may also have potential adverse effects on glucose metabolism, wound healing, and infection [119]. As a result, the use of corticosteroids is summarized as a Class IIb recommendation by the SCA/EACTA 2019 Practice Improvement Advisory for AF prophylaxis. So far, corticosteroids are not widely used for the purpose of prevention of AFACS.

##### Non-steroidal Anti-inflammatory Drugs

Transient interest in the use of non-steroidal anti-inflammatory drugs (NSAIDs) started with a single-center 2004 RCT that randomized 100 patients undergoing CABG to an NSAID regimen (of intravenous ketorolac in the immediate perioperative period followed by oral ibuprofen) vs placebo [77]. These authors found the NSAID group had significantly reduced AFACS (9.8% vs 28.6%, *p* = 0.017) without any difference in renal failure [77]. A subsequent RCT of CABG patients comparing naproxen vs placebo discontinued enrollment early, due to a significantly higher rate of renal failure in the naproxen group. These authors found no significant reduction in AFACS in the naproxen group amongst the 161 (out of an intended 200) enrolled patients (15.2% vs 7.3%, *p* = 0.11) [78] but may have been underpowered to detect a statistically significant difference. In addition to the risk of renal failure, the use of NSAIDs in cardiac surgery is limited by a potential risk of bleeding, and particularly with the COX-2 inhibitors, myocardial ischemia, or infarction [116]. With the lack of RCTs and the adverse effect profile, NSAIDs are not used routinely for preventing AFACS and have not been addressed by guidelines.

##### Colchicine

Colchicine also has potent anti-inflammatory properties [113], and its role in AFACS prevention was addressed by multiple meta-analyses [79–82]. The most recent meta-analysis included 5 RCTs (*n* = 1412), and the authors reported that patients receiving colchicine had a significant reduction in AFACS when compared with those receiving placebo (18% vs 27%, risk ratio 0.69, 95% CI 0.57–0.84, *p* = 0.0002) and a 1.2 day decrease in hospital length of stay but no significant change in major adverse events [83]. Gastrointestinal intolerance was the main adverse effect. With its relatively good adverse effect profile and growing, but not conclusive, evidence to support efficacy as primary prevention, the SCA/EACTA Practice Advisory summarizes a Class IIb recommendation for colchicine in AFACS prevention although it is not used widely for this purpose.

#### Antioxidant agents

##### Statins

The theorized mechanism of action of statins is multifactorial [113], and there are numerous studies using statins as an intervention in cardiac surgical patients. Two meta-analyses were published in 2016: One specifically included RCTs of statin-naïve patients who were randomized to statin vs placebo and found that statin therapy was associated with a significant reduction in AFACS (RR 0.50, 95% CI 0.41–0.61, *p* < 0.0001) [128]. The other found only two trials with low risk of bias that reported atrial fibrillation as an outcome and concluded that there was no difference in the rate of AFACS in statin vs placebo groups (25.07% vs 23.6%, OR 1.08, 95% CI 0.9–1.3, *p* = 0.40) [84]. Conversely, a 2014 meta-analysis found that patients on statins had a 32% reduced risk of new-onset AFACS compared with controls, after adjusting for publication bias (OR 0.68; 95% CI 0.54–0.85) [85]. This series of conflicting studies is representative of preceding reviews and meta-analyses. The routine use of statins in AFACS prophylaxis remains controversial, but a high proportion of adult patients undergoing cardiac surgery will already have an indication for statin use, and it is rare that there is a reason to stop these agents perioperatively.

##### Polyunsaturated Fatty Acids

Polyunsaturated fatty acids (PUFAs) are a dietary antioxidant with possible benefits to overall cardiovascular morbidity shown in animal models. There is limited evidence for their use in AFACS prophylaxis [119]. Early trials have not shown a reduction in AFACS [113], and a 2014 meta-analysis of 8 RCTs (*n* = 2687) concluded that preoperative PUFA treatment did not influence AFACS incidence [86]. In 2017, a meta-analysis of 19 RCTs (*n* = 4335) found a reduction in AFACS [87], and so did a 2018 meta-analysis that included 14 RCTs (*n* = 3570), which found significant AFACS reduction with PUFA vs controls (OR 0.84, 95% CI 0.71–0.99, *p* = 0.03), although this effect was found only in CABG and not valve surgery [88]. This appears to be a promising intervention for AF prophylaxis, which may incur few risks from adverse effects and costs, but has not been addressed by any guidelines to date.

##### Levosimendan

While levosimendan was not introduced for its current indications in heart disease as an antioxidant, some authors have proposed that its antioxidant properties could help in AFACS prophylaxis [113]. Levosimendan works to augment myocardial inotropy by increasing myofilament sensitivity to calcium without increasing myocardial oxygen consumption [113, 129]. There is conflicting evidence about whether the use of levosimendan protects against or predisposes to AFACS: 1 RCT of 200 patients whose primary endpoint was AFACS found a decrease in the levosimendan group compared to controls (12% vs 36%, *p* < 0.05) [130]. All other studies have evaluated AFACS as a secondary outcome, and meta-analyses have found either no difference [92] or an increase [131] in AFACS in patients receiving levosimendan. It is unlikely that levosimendan will be ever used solely for the purpose of AFACS prophylaxis.

##### N-Acetylcysteine

N-Acetylcysteine (NAC) has free radical scavenging and antioxidant properties and has showed promising results in two meta-analyses [93, 94]. A meta-analysis carried out in 2014 included 10 RCTs (*n* = 1026), 8 of which administered NAC intravenously, and 2 orally, and found a reduction in AFACS incidence when compared to controls (OR 0.56; 95% CI 0.4–0.77, *p* < 0.001) and all-cause mortality (OR 0.40, 95% CI 0.17–0.93, *p* = 0.03) without a difference in the cerebrovascular events, ICU or hospital stay [94]. In 2016, a further meta-analysis found the same 10 RCTs (*n* = 1026) that reported AFACS as an outcome and it reported the same finding [89]. A recent RCT of 150 patients undergoing on-pump CABG randomized to 50 mg/kg IV NAC or placebo found a significant decrease in AFACS (5.6% vs 18.8%, OR 0.23, 95% CI 0.08–0.82, *p* = 0.02) [132]. N-Acetylcysteine appears promising and has a few adverse effects, but has not been addressed specifically by any guidelines for routine use.

##### Vitamin C

Vitamin C is another agent that may reduce oxidative stress and has been studied in a few small trials for AFACS prophylaxis. A 2016 meta-analysis of 7 RCTs (*n* = 785) found a reduction in AFACS in patients randomized to vitamin C vs placebo (OR: 0.40, 95% CI 0.23–0.68, *p* = 0.001) [89]. More recently, an RCT of 314 on-pump CABG patients found no difference in AFACS, ICU or hospital lengths of stays [133]. No guidelines currently reference its use for AF prophylaxis.

##### Combined Antioxidants

A 2013 study randomized 203 cardiac surgical patients to a combined regimen of Vitamins C, and E and PUFAs. The authors found a significantly reduced incidence of AF in the patients receiving antioxidants compared to controls (9.7% vs 32%, RR 0.28, 95% CI 0.14–0.56, *p* < 0.001). The authors suggest that the simultaneous use of these antioxidants has potential for being effective, safe, and low-cost AFACS prophylaxis [134]. However, no guidelines currently reference such a protocol.

#### Vasopressor Agents

The recent Vasopressin vs Norepinephrine in Patients with Vasoplegic Shock after Cardiac Surgery (VANCS) trial found that using vasopressin to treat vasoplegia after cardiac surgery—compared to norepinephrine—was associated with a lower occurrence of atrial fibrillation (63.8% vs 82.1%; *p* = 0.0004) [100]. The composite end point of 30-day mortality or severe complications was also decreased in the vasopressin group (32% vs 49% unadjusted hazard ratio, 0.55; 95% CI, 0.38–0.80; *p* = 0.0014) [100]. The authors suggest this may be the result of reduced beta-1 receptor stimulation in the atrial myocardium by a non-catecholaminergic agent. Although these data might inform a choice between vasopressor agents, it is unlikely that vasopressin will ever be used solely for the purpose of AFACS prophylaxis.

#### Electrophysiological or Surgical Strategies

##### Atrial Pacing

The use of prophylactic overdrive atrial pacing after cardiac surgery improves intra-atrial conduction and prevents triggering events such as premature atrial contractions or atrial refractoriness [13, 114]. Multiple studies report favorable results with the use of right atrial pacing, left atrial pacing, Bachmann’s bundle pacing, and bi-atrial pacing [49, 52, 113, 135, 136]. A 2013 Cochrane database review and meta-analysis including 21 RCTs (*n* = 2933) found a significantly decreased AFACS incidence in all pacing groups (18.7% in pacing groups and 32.8% in control groups, OR 0.47, CI 0.36–0.61, *I*^2^ = 50%) [49]. It is less clear whether the site of pacing has a further impact on efficacy. An earlier 2006 meta-analysis of 14 RCTs reported that a significant reduction in AFACS occurred with bi-atrial pacing but not with single-site right or left atrial pacing alone [52]. Other authors have suggested that epicardial pacing could have pro-arrhythmic properties [137] and that right atrial pacing is the most effective site for preventing AFACS [114, 138]. Routine use of prophylactic pacing is limited by the potential risks associated with placement or removal of temporary pacing wires, such as mediastinal infection and damage to coronary grafts or atriotomy sites resulting in tamponade [116]. The use of atrial pacing has been summarized by the SCA/EACTA Practice Advisory as Class IIb for the prophylaxis of AF.

##### Posterior Pericardiotomy

A posterior pericardiotomy allows pericardial fluid to drain out of the pericardial space, thus decreasing the accumulation of pericardial effusions, which may be a trigger for atrial fibrillation and supraventricular tachyarrthythmias [101]. Three meta-analyses have been published: a 2010 meta-analysis of 6 RCTs (*n* = 763) found that there was a significant decrease in AFACS in the posterior pericardiotomy group when compared with that in the control group (10.8% vs 28.1%, *p* = 0.003, OR 0.33, 95% CI 0.16–0.69) [101]. A 2013 meta-analysis found the same 6 published RCTs, and similarly reported a significant reduction in AFACS [49]. In 2016, a meta-analysis identified 10 RCTs (*n* = 1648) and also found that patients receiving a posterior pericardiotomy had a decreased incidence of AFACS (10.6% compared with 24.9% in controls, *I*^2^ = 55%, *p* < 0.00001, OR 0.36, 95% CI 0.23–0.56) [102]. There appears to be consistent evidence from this small number of studies for using a posterior pericardiotomy as a prevention strategy for AFACS, although this has not been conclusively proven in an adequately powered study and has not been addressed by any society guidelines to date. While this is a relatively simple intervention with favorable data, potential risks that authors have pointed out include the potential for compression of bypass grafts or cardiac herniation [101, 102].

##### Epicardial Fat Pad Manipulations

The autonomic nervous system may contribute to AFACS susceptibility, as atrial tissue receives extensive cholinergic innervation, and an enhanced vagal tone results in decreased atrial refractoriness [103, 139]. Vagal postganglionic neurons are located in distinct anatomic fat pads distributed around the heart, including the anterior epicardial fat pad, and interventions targeting these neurons were hypothesized to have an effect on AFACS. To date, these interventions are not referenced for AFACS prophylaxis in any guidelines.

##### Anterior Fat Pad Preservation vs Dissection or Removal

Dissecting the epicardial fat pad to reveal an aortopulmonary window for aortic cannulation and cross-clamp placement is a routine step in cardiac surgery. Some authors have hypothesized that the disruption [103] or removal [104] of the anterior fat pad might be useful in decreasing AFACS. However, in a study of 55 patients undergoing CABG, the incidence of AFACS was significantly lower in the group randomized to anterior fat pad preservation than the group with anterior fat pad dissection (7% vs 37%, *p* < 0.01) [103]. Contradictory results from a study of 180 patients concluded that preserving the anterior fat pad did not reduce AFACS [105]. A 2015 meta-analysis that included 7 RCTs (*n* = 991) concluded that the removal of the anterior fat pad did not lead to a decreased risk of AFACS, but did not examine the question of whether the converse was true—that is, whether preserving the fat pad would influence AFACS risk [104]. Further studies are required before a recommendation can be made about how surgical manipulation of the anterior fat pad might affect AFACS.

##### Fat Pad Botulinum Toxin Injection

The protein botulinum toxin (BTX) prevents the release of the neurotransmitter acetylcholine from axon endings at the neuromuscular junction, and this suppression of vagal tone has been found to reduce AF in multiple animal models. A prospective, randomized, double-blind study of 60 patients in 2014 found promising results: CABG patients with a history of paroxysmal but not persistent or permanent forms of AF who received BTX injections into four major epicardial fat pads prior to aortic cross-clamp release had a lower incidence of AFACS than those receiving normal saline (7% vs 30%, *p* = 0.024) [106]. Amongst the patients who had AFACS, the AF burden was also lower in the BTX group (0.3% vs 2.5%, *p* = 0.08) [106], and at 1-year follow-up recurrent atrial fibrillation was also significantly lower in the BTX group (27% vs 0%, *p* = 0.002). In their 3-year follow-up, the BTX group still had decreased incidence of AF (23.3% vs 50% in placebo group, hazard ratio 0.36, 95% CI 0.14–0.88, *p* = 0.02) [107]. However, another RCT of 130 patients where only 4 patients had a history of AF found a trend that failed to reach statistical significance (36.5% vs 47.8% in placebo, *p* = 0.18, absolute risk reduction of 11%) [109]. This suggests that the effect size of BTX fat pad injections might be smaller in AF-naïve patients and it may be a more useful intervention for decreasing AFACS incidence in patients who already have a history of paroxysmal AF.

##### Concomitant Surgical Ablation

Preoperative AF has been identified as a risk factor for AFACS [140], and AF ablation surgery has been shown to improve outcomes in patients with paroxysmal AF undergoing cardiac surgery [141–144], although one of these studies also reported that at 1-year follow-up, there was no difference in the quality of life [145]. There are no available data about whether AFACS is reduced by the prophylactic intraoperative ablation in patients without a history of AF [113, 146], and this issue has not been addressed in the guidelines.

##### Off-pump Coronary Artery Bypass Grafting

The inflammatory response to cardiopulmonary bypass (CPB) has been identified as a potential contributor to AFACS. Therefore off-pump CABG, which has been found to have a decreased inflammatory response, could in theory decrease AFACS [113, 147]. However, there is little evidence that off-pump CABG decreases AFACS incidence when compared to on-pump CABG. An analysis of the “Randomized On Versus Off Bypass” trial, which included 2103 patients, found no increase in the rate of AFACS in on-pump vs off-pump CABG [148]. A 2012 meta-analysis of 34 trials (*n* = 3392) showed that although there may be a significant intervention effect in favor of off-pump CABG (RR 0.86; 95% CI 0.76–0.96, *p* = 0.008), no significant difference was found when only trials with low risk of bias were included [110]. More recently, the randomized controlled multicenter German Off-Pump CABG in the Elderly trial of 2303 patients also found the same rate of AFACS in both groups [111]. In the ongoing debate about the merits of on-pump vs off-pump CABG, the influence of either strategy on the incidence of AFACS is unlikely to tip the scales.

## Treatment Strategies and Associated Evidence Base

The treatment strategies for AFACS depend on hemodynamic stability and clinical symptoms. The approach to a hemodynamically stable patient is broadly classified into rate control, rhythm control, and concomitant thromboprophylaxis [113]. These treatment strategies and the associated strength of society recommendation, if available, are summarized in Table 2.

### Rate vs Rhythm Control

Available data suggest that most cases of AFACS return to a sinus rhythm at the end of 24 hrs regardless of treatment strategy [113, 123, 149]. An RCT published in 2000 randomized 50 cardiac surgery patients with new-onset AF to rate vs rhythm control and found no difference in the time of conversion to sinus rhythm (11.8 ± 3.9 hrs vs 11.2 ± 3.2, *p* = 0.8) and no difference in relapse rates for the 2-month follow-up duration, although hospital length of stay was reduced in the anti-arrhythmic arm [149].

Rate control is generally the recommended strategy by available guidelines that address AFACS [113, 149, 150] for hemodynamically stable patients within the first 24 hrs of AF. A multicenter trial randomized 523 cardiac surgical patients to rate control (ventricular rate less than 100 bpm) vs rhythm control (amiodarone followed by electrical cardioversion if AF persisted for 24–48 hrs) [151]. There was no detectable difference in the proportion of patients who were free from atrial fibrillation in the rate vs rhythm control groups at 30 days (84.2% vs 86.9%, *p* = 0.41) or 60 days (93.8% vs 97.9%, *p* = 0.02) after discharge [151]. There was also no difference between the groups in hospital length of stay, rates of death, and overall adverse events including thromboembolic and bleeding events [151]. However, there was a 25% crossover between groups. The authors note that rate control strategies avoided many side effects of rhythm control drugs and did not make a significant difference in postoperative outcomes [151]. This large study has led some authors to suggest that in cardiac surgical patients, new-onset AF is a self-limiting disease which often resolves regardless of its initial treatment [152]. The overall impact of increased AF burden (frequency and duration of AF) in AFACS has not been conclusively understood.

The most frequently used agents for rate control are beta-blockers and non-dihydropyridine calcium channel blockers, and these can potentially be used in combination [113]. A variety of target ventricular rates, from 80–110 bpm, have been proposed [106]. The cumulative effects of drugs given for controlling the ventricular response rate in AF can cause problematic bradycardia in the event of spontaneous cardioversion to sinus rhythm.

SCA/EACTA, ESC, and ACC/AHA/HRS guidelines all include Class IIa recommendations for managing asymptomatic patients with rate control and anticoagulation, with ACC/AHA/HRS guidelines also recommending cardioversion if the AF does not spontaneously revert to sinus rhythm during sub-sequent follow-up [123].

#### Digoxin

While digoxin does not decrease the incidence of AFACS [113], and thus it is not recommended for prophylaxis, the EHRA/EACPR/HRS/APHRS [119] and ACC/AHA/HRS [59, 123] guidelines describe its treatment role for rate control in the management of patients with rapid ventricular responses. Digoxin has a slower onset of action and may be less effective in the setting of high catecholaminergic states, for instance in postoperative patients [113]. One RCT assessing AFACS reported significantly fewer patients achieved rate control with digoxin when compared to diltiazem at 2 hrs (75% vs 35%, *p* = 0.03) and 6 hrs (85% vs 45%, *p* = 0.02) following drug administration; however, the 12- and 24-hr response rates were similar [153]. When used together with other rate control agents, digoxin may have a dose-sparing effect for concomitant beta-blocker or calcium channel blocker, and this potentially avoids some degree of hypotension [113]. However, digoxin is also contraindicated in patients with significant renal impairment or an accessory pathway [113].

##### Rhythm Control Interventions

Paroxysmal AF is associated with electrical and structural remodeling of the heart, which has been identified as a cause of progression to persistent AF [113, 119]. ESC and ACC/AHA/HRS practice guidelines state that it is reasonable to restore sinus rhythm pharmacologically with ibutilide or direct-current cardioversion in patients who develop AFACS, or to administer antiarrhythmic medications in an attempt to maintain sinus rhythm in recurrent or refractory AFACS [59, 123, 150]. In patients at a risk of postoperative bleeding, it has been suggested that rhythm control may also help to avoid the need for anticoagulation therapy that is conventionally indicated for AFACS lasting longer than 48 hrs [113].

#### Electrical Cardioversion

R-wave synchronized direct-current electrical cardioversion (DCCV) is indicated for AFACS patients with hemodynamic instability, or with evidence of acute myocardial ischemia or infarction [5, 106, 113]. If restoration of sinus rhythm is attempted more than 48 hrs after the onset of AFACS, exclusion of intracardiac thrombus (most commonly seen in the left atrial appendage) and/or anticoagulation should be considered prior to electrical or chemical cardioversion [113], as discussed in further detail below. If there is a need for repeat cardioversion, concurrent pharmacologic rhythm or rate-control drugs can be considered to optimize successful and sustained cardioversion [113]. To our knowledge, no comparison of the effectiveness of electrical vs chemical cardioversion has been undertaken. It has been reported that only 1 in 10 patients with AFACS receive electrical cardioversion compared with three quarters who receive amiodarone [11].

#### Pharmacologic Cardioversion

The mechanism of action of antiarrhythmic agents involves some degree of interference with myocyte sodium and/or potassium channels. Other than amiodarone and beta-blockers (discussed previously), the available antiarrhythmic agents include vernakalant, ibutilide, flecainide, propafenone, dronedarone, disopyramide, and quinidine [113]. None of them have been studied specifically in the setting of cardiac surgical patients, and practice guidelines are based on evidence from other AF populations.

##### Ibutilide

In the ACC/AHA/HRS practice guidelines, ibutilide is specifically named as a reasonable choice of pharmacological agent for restoring sinus rhythm in AF [123]. It is associated with ventricular arrhythmias including sustained polymorphic ventricular tachycardia, requires close rhythm monitoring for at least 4 hrs after administration, and it is contraindicated in patients with QT prolongation, hypokalemia, and reduced ejection fractions [113, 154].

##### Vernakalant

Vernakalant was approved in 2010 by the European Medicines Agency for the cardioversion of new-onset AF of 3 days or less in cardiac surgical patients and also for the cardioversion of other AF less than 7 days in duration. There have been 4 RCTs evaluating the efficacy of vernakalant in new-onset AF, three of which compared the drug against placebo [155–157] and one superiority study with amiodarone in the control group [158]. Only one of these specifically recruited cardiac surgical patients with new AF, and these authors found that vernakalant converted 47% of patients to sinus rhythm within 90 min, compared with 14% of patients receiving placebo (*p* < 0.001) [155]. All reported that vernakalant was safe and when compared with placebo, resulted in rapid conversion to sinus rhythm within 90 min [155–158]. The ESC guidelines contain a Class IIb recommendation to consider vernakalant for cardioversion of POAF in patients without severe heart failure, hypotension, or severe structural heart disease, in particular aortic stenosis [150]. It also prolongs the QT interval; however, none of the 4 RCTs reported torsades de pointes, polymorphic, or other sustained ventricular tachycardia [155–158]. A recent observational study that characterized its use in post-cardiac surgical patients found that 44% of patients who received vernakalant converted to sinus rhythm after one 3-mg/kg dose, and another 32% converted after a second dose of 2 mg/kg, with a mean time to conversion of 13.7 ± 14.1 min [159]. These authors also reported that patients receiving vernakalant had a decreased conversion rate if they had no preoperative beta-blocker, postoperative troponin levels > 500 ng/ml, and systolic blood pressures > 140 mmHg and if they had undergone valve surgery (as opposed to isolated CABG) [159]. At their first follow-up clinic visit after discharge, 92% of responders were in sinus rhythm, compared with 80% of non-responders (*p* < 0.01) [159]. Vernakalant has not been approved in the USA but is recommended by European societies (refer to Table 2) for the pharmacologic conversion of POAF.

### Electrophysiological or Surgical Interventions

In patients with persistent forms of AF, interventions such as catheter ablation, or atrioventricular nodal ablation with permanent pacemaker implantation, may help to restore regular ventricular rhythm [113]. However, as discussed above, many AFACS patients return to a sinus rhythm in time and these interventions are not typically utilized in the immediate post-operative setting [106, 151].

### Anticoagulation

Current recommendations from multiple societies (refer to Table 2) indicate that it is reasonable to consider antithrombotic therapy for AFACS lasting more than 48 hrs or of an unknown duration, as advised for nonsurgical patients [123, 150]. AF is a risk factor for thromboembolic events including thromboembolic stroke, and stroke risk can be assessed by scoring systems such as the CHADS2 [160] or CHADS2VASC [35] score. Any antithrombotic therapy also increases bleeding risk, which must be carefully considered particularly, in the immediate postoperative period, and scoring systems for this have been developed in the form of the HAS-BLED [161], ATRIA [162], and HEMORR2HAGES [163] scores, albeit not specifically in the setting of cardiac surgery. It remains uncertain whether AFACS conveys the same thromboembolic risk as AF occurring outside of this context. It has also been reported that the CHA2DS2VASC score can predict postoperative stroke risk, independent of the presence of AF [164]; delayed postoperative strokes are traditionally attributed to postoperative AF but these data do not support this concept.

The long-term prognosis of AFACS that reverts early to sinus rhythm is also uncertain. Studies of AF in patients admitted to intensive care units for sepsis have shown an increased lifetime risk with increasing AF burden during the critical care episode [165].

#### Anticoagulation and Cardioversion

Cardioversion also poses a risk of stroke in non-anticoagulated patients [150]. Prior to cardioversion in patients with AF lasting more than 48 hrs or of unknown duration, antithrombotic therapy or transesophageal echocardiography (TEE) should be carried out to exclude the presence of any intracardiac thrombus, particularly in the left atrial appendage [166, 167]. TEE imaging might be useful to facilitate safe cardioversion for postoperative patients in whom bleeding risk is high. ESC guidelines state that in patients with an identified thrombus, cardioversion should not be performed until at least 3 weeks of anticoagulation therapy has been achieved, and anticoagulation should be continued for 4 weeks after if there is no other indication for long-term anticoagulation [150].

## Areas for Further Investigation

As described above, there is a paucity of definitive data in the form of appropriately powered randomized controlled trials to determine whether a successful reduction in AFACS burden by means of the above prevention and treatment strategies translates into a meaningful decrease in adverse outcomes, such as cerebrovascular events or other thromboembolic events, morbidity from anticoagulation, mortality, critical care or hospital lengths-of-stays, or quality of life. Unfortunately, as eloquently noted by Sessler in his recent call to action [168], this lack of adequately sized trials providing clinically actionable results is not limited to AFACS investigations but is widespread across many disciplines.

It remains uncertain whether prophylactic interventions to prevent AFACS should be limited only to high-risk patients or whether all patients should receive at least some of these interventions.

The only recent meta-analysis analyzing multiple interventions for the prevention of AFACS found that no individual intervention was associated with a significant effect on postoperative mortality, and the authors acknowledged that they were significantly limited by the lack of relevant secondary outcome data [49]. One possibility for the dearth of effective AFACS interventions is the fact that many attempted strategies have been developed from extrapolated data from patients with primary AF. While the initiation of AF in the primary, non-surgical setting and the secondary, postoperative setting is both multifactorial in etiology and likely to have some amount of mechanistic overlap, there may be causes of a susceptible substrate and triggering factors that are more specific to the primary or secondary context. For example, during the immediate postoperative period, factors such as myocardial ischemia/reperfusion injuries, the presence of direct surgical injury, rapid fluid shifts with electrolyte changes, and use of exogenous inotropic agents create a markedly different physiologic environment than that seen surrounding primary AF. Further, there is signal in the data that suggests there is more than one phase of AF risk in the postoperative period [26] consistent with the idea that multiple sets of predisposing/triggering mechanisms may be at play. Similarly, some interventions used in the primary AF setting have a very different risk:benefit ratio when considered in the postoperative period, for example, the initiation of anticoagulation. A deeper understanding of post-cardiac surgery-specific factors predisposing, triggering, and sustaining AFACS will allow for more targeted trials examining prevention strategies and treatment priorities for this patient group.

## Conclusions

AFACS is the most common adverse event after cardiac surgery and is associated with significant morbidity, mortality, and cost. It is unlikely that there is a single unifying mechanism for development of this arrhythmia and current studies point to the high likelihood of multiple disparate pathways leading to the common outcome of AFACS. Supported by studies with high levels of evidence, multiple societal guidelines have made recommendations supporting the use of prophylactic and treatment interventions. It remains uncertain whether the relationship between AFACS and poorer outcomes is causative. Well-designed future studies in the field should aspire to clarify the effects of their short-term interventions on longer-term outcome measures.

Development of a validated risk-stratification model would help to appropriately target protocols for prevention of AFACS, minimizing some of the non-trivial risks of either AFACS itself, or routinely used preventative measures.

## Figures and Tables

**Table 1:** Strategies for prevention of AFACS

AFACS prophylaxis			

	Strategy	Level of evidence	Society recommendations
Pharmacologic prophylaxis strategies	Magnesium supplementation	Level I—intraoperative magnesium administration is associated with decreased AFACS [49].	None
	Potassium supplementation	Practice surveys—common practice to replete potassium during the perioperative period for a target serum level of 4.5–5.5 mEq/L [50, 51].	None
	Beta-adrenergic blockers	Level I—perioperative use is associated with decreased AFACS [49, 52–58].	Class I—[47, 48, 59–61] specifically recommends the administration of beta-blockers for at least 24h in patients with no contraindications.
	Amiodarone	Level I—perioperative use reduces incidence of AFACS; useful in patients at high risk [49, 62–67].	Class IIa—[47, 48, 59, 60] Class IIa—[61] recommends use as a second-line agent to prevent AFACS when beta-blockade is contraindicated.
	Sotalol	Level I—perioperative use reduces incidence of AFACS; however, there is a risk of significant bradycardia and ventricular arrhythmias [49, 52, 68–70].	Class IIb—can be considered for patients at high risk for AFACS. [47, 48, 59, 71] states that it has limited utility due to adverse effects.
	Ranolazine	Level I—perioperative use reduces AFACS; however, larger randomized trials are needed [72, 73].	None
	Non-dihydropyridine calcium channel blockers	None—commonly used for treatment of AFACS but has not shown promise as a prophylactic agent.	None
	Digoxin	None—commonly used for treatment of AFACS but has not shown promise as a prophylactic agent.	None
	Corticosteroids	Level I—authors of a recent meta-analysis cautioned that only small trials found an effect [74–76].	Class IIb—the type and dose of an effective corticosteroid remains to be established [47, 48].
	NSAIDs	Conflicting level 1—use will also be limited by risks of renal failure, bleeding, and myocardial ischemia [77, 78].	None
	Colchicine	Level I—reduction in recurrence of atrial fibrillation after cardiac surgery or pulmonary vein isolation procedures [79–83].	Class IIb–[47, 48, 59]
	Statins	Highly conflicting Level I—regarding association with AFACS [84, 85].	None
	PUFAs	Level I—significant reduction noted in AFACS [86–90].	None
	Levosimendan	Conflicting level I—one meta-analysis found decreased AFACS [91]; however, another did not[92].	None
	N-Acetylcysteine	Level I—significant reduction in AFACS with IV or PO administration [89, 93–95].	None
	Vitamin C	Level I—meta-analyses of small trials found a reduction in AFACS [89, 96–99].	None
	Vasopressin vs norepinephrine	Level II—use of vasopressin intraoperatively or in the immediate postoperative period is associated with decreased AFACS compared to norepinephrine [100].	None
Surgical prophylaxis strategies	Atrial pacing	Level I—the prophylactic use of atrial pacing after cardiac surgery is associated with significantly decreased AFACS [49].	Class IIb—optimal pacing site(s) not specified [47, 48].
	Posterior pericardiotomy	Level I—significant reduction in AFACS in patients who receive a posterior pericardiotomy compared with controls [49, 101, 102].	None
	Anterior fat pad preservation	Conflicting level II—whether preserving the anterior fat pad decreases AFACS [103–105].	None
	Botulinum toxin (BTX) injection	Conflicting level II—for whether injecting BTX into the epicardial fat pads decreases AFACS [106–109].	None
	Off-pump CABG	Level I—meta-analyses have found no effect of on-pump vs off-pump CABG in AFACS [110, 111].	None
	Concomitant surgical ablation	None—may be used in patients with existing atrial fibrillation; however, there is no evidence for whether it is useful as a prophylactic strategy.	None

**Table 2:** Strategies for treatment of AFACS

AFACS treatment			
	Strategy	Level of evidence	Society recommendations
Rate control	Beta-blockers	Level II—most commonly used are esmolol and metoprolol [122].	Class I—is recommended as a first-line agent for rate control[47, 48, 59].
	Non-dihydropyridine calcium channel blockers	Level II—verapamil and diltiazem can be used in patients who have contraindications to beta-blockers, or in conjunction with beta-blockers [122].	Class I—is recommended to use as a second-line agent after beta-blockers [47, 48, 59].
	Digoxin	None. Delayed rate control in digoxin compared to diltiazem at 2 hrs after administration[122].	Not specifically addressed
	Amiodarone	Level II/III—also has rhythm control properties, and is more effective at maintaining sinus rhythm when compared with dronedarone, sotalol, flecainide, and propafenone [122].	Class IIa—[47, 48].
Rhythm control	Electrical cardioversion	Level III—R-wave synchronized direct-current electrical cardioversion is indicated in hemodynamically unstable patients, or with evidence of myocardial ischemia, or infarction [113].	Class IIa—it is reasonable to restore sinus rhythm pharmacologically with ibutilide or direct-current cardioversion in patients who develop AFACS, or to administer antiarrhythmic medications in attempt to maintain sinus rhythm in recurrent or refractory AFACS [47, 48, 59, 60].
	Ibutilide sotalol	None—have not been specifically studied in the setting of cardiac surgery. Use with caution in QT prolongation, hypokalemia, and reduced ejection fractions [60].	
	Vernakalant	None—may be used for cardioversion of AFACS in patients without severe heart failure, hypotension, or severe structural heart disease, in particular aortic stenosis [60].	Class IIb—[60].
Anticoagulation	Anticoagulation	Antithrombotic therapy should be considered for AFACS lasting ≥ 48 hrs or of unknown duration [60].	
	For cardioversion	Prior to cardioversion of AF ≥ 48 hrs or of unknown duration, TEE should be considered to rule out intracardiac thrombus or cardioversion should take place only after 3 weeks of anticoagulation therapy has been achieved, after which, anticoagulation should be maintained for 4 weeks after; there is no further indication for continued antithrombotic therapy[60].	

Society guidelines [47, 48] 2019 SCA/EACTA Practice Advisory for the Management of Perioperative Atrial Fibrillation in Patients Undergoing Cardiac Surgery [71] 2017 EHRA/EACPR/HRS/APHRS Position Paper on How to Prevent Atrial Fibrillation [60] 2016 ESC/EACTS Guidelines for the Management of Atrial Fibrillation [59] 2011 ACCF/AHA/HRS Focused Updates of the Guidelines for the Management of Patients with Atrial Fibrillation [61] 2011 ACCF/AHA Guideline for Coronary Artery Bypass Graft Surgery
